# Corticospinal Tract Change during Motor Recovery in Patients with Medulla Infarct: A Diffusion Tensor Imaging Study

**DOI:** 10.1155/2014/524096

**Published:** 2014-05-25

**Authors:** Dongdong Rong, Miao Zhang, Qingfeng Ma, Jie Lu, Kuncheng Li

**Affiliations:** ^1^Department of Radiology, Xuanwu Hospital of Capital Medical University, Beijing 100053, China; ^2^Beijing Key Laboratory of Magnetic Resonance Imaging and Brain Informatics, Beijing 100053, China; ^3^Department of Neurology, Xuanwu Hospital of Capital Medical University, Beijing 100053, China

## Abstract

Diffusion tensor imaging (DTI) and tractography (DTT) provide a powerful vehicle for investigating motor recovery mechanisms. However, little is known about these mechanisms in patients with medullary lesions. We used DTI and DTT to evaluate three patients presenting with motor deficits following unilateral medulla infarct. Patients were scanned three times during 1 month (within 7, 14, and 30 days after stroke onset). Fractional anisotropy (FA) values were measured in the medulla, cerebral peduncle, and internal capsule. The three-dimensional corticospinal tract (CST) was reconstructed using DTT. Patients 1 and 2 showed good motor recovery after 14 days, and the FA values of their affected CST were slightly decreased. DTTs demonstrated that the affected CST passed along periinfarct areas and that tract integrity was preserved in the medulla. Patient 3 had the most obvious decrease in FA values along the affected CST, with motor deficits of the right upper extremity after 30 days. The affected CST passed through the infarct and was disrupted in the medulla. In conclusion, DTI can detect the involvement and changes of the CST in patients with medulla infarct during motor recovery. The degree of degeneration and spared periinfarct CST compensation may be an important motor recovery mechanism.

## 1. Introduction

Lateral medulla infarction occurs fairly infrequently in neurology clinical practice. Patients often present with limited neurologic deficits and, on occasion, hemiparesis accompanies medulla infarction. The corticospinal tract (CST) is the major neuronal pathway that mediates voluntary movements in humans. Many studies have elucidated the motor recovery mechanisms following ischemic stroke associated with the integrity of the CST. However, most previous studies have focused on the corona radiata and pontine lesions. Little is known about the motor recovery mechanisms in patients with medullary infarction.

Magnetic resonance imaging (MRI) plays an important role in the diagnosis and treatment of acute stroke. With the advent of diffusion-weighted imaging (DWI) techniques, small infarctions occurring in the medulla can be more easily identified. Furthermore, diffusion tensor imaging (DTI) can evaluate the degree of fiber damage in stroke affecting the CST. Diffusion tensor tractography (DTT), derived from DTI, allows for visualization of the architecture and integrity of the CST in three dimensions and can assess white matter tracts, such as the CST, at the subcortical level. The validity and reliability of DTT for CST have been demonstrated in previous studies [1–5]. In this study, we report three patients who showed hemiparesis due to isolated unilateral medulla infarct. We used DTI to investigate the involvement and change of the CST during motor recovery.

## 2. Materials and Methods 

### 2.1. Participants

Three right-handed patients with isolated unilateral medulla infarct (a 55-year-old man, a 54-year-old man, and a 74-year-old woman) were recruited for this study. The study protocol was approved by the local Institutional Review Board and written informed consent was obtained from all participants.

We recruited the three stroke patients using the following enrollment criteria: having first-onset stroke and manifested motor deficits, fully obtained admission history (within 7 days after onset of symptoms), single infarction confined to the medulla identified on MRI, and no other concomitant brain lesion or previous infarcts. The exclusion criteria were as follows: contraindications for MRI, unclear onset of symptoms, lesions outside the medulla, recurrence infarction during followup, deafness and/or blindness, aphasia, or a visual field deficit. All patients were scanned three times during a period of 1 month (within days 7, 14, and 30 after stroke onset).

### 2.2. MRI Data Acquisition

MRI scanning was performed on a 3.0-Tesla whole-body scanner (Trio Tim, Siemens). Traditional axial T1-weighted (TR 155 ms/TE 2.81 ms), fast-spin echo T2-weighted imaging (TR 3830 ms/TE 98 ms), FLAIR (TR 8500 ms/TE 87 ms), and DWI (TR 3000 ms/TE 91 ms; *b* = 0, 500 and 1000 s/mm^2^) were performed. The DTI acquisition parameters were as follows: TR 8000 ms/TE 83 ms, NEX = 1, matrix 128 × 128, field of view 24 × 24 cm, *b* = 0, 700 s/mm^2^, and a slice thickness of 2 mm without a gap. We acquired 64 contiguous slices parallel to the anterior commissure-posterior commissure line.

### 2.3. DTI Data Postprocessing

DTI data were transferred to a work station (Multi-Modality Work Place, Siemens Healthcare) for processing. Circular regions of interest (ROI) were symmetrically drawn on axial slices on the left and right sides along the pyramidal tract pathway at three levels: the medulla, cerebral peduncle, and posterior limb of the internal capsule along the CST. To include only the CST region, the ROI size was set between 30 mm^2^ and 35 mm^2^ voxels. FA values for each ROI were obtained by averaging all voxels within the ROI. The FA ratio (rFA) between the infarct ipsilateral and contralateral side was calculated (rFA = FA_ipsilateral  side_/FA_contralateral  side_) in each patient. The three-dimensional CST was reconstructed using Siemens software. For fiber tracking of the CST, two ROIs were manually placed on two-dimensional transverse color-coded directional FA images. The upper ROI was placed on the posterior limb of the internal capsule and the lower ROI was placed on the lower pons. Only the fibers passing through both ROIs were displayed and designated as the CST. The thresholds of the tracking termination were 0.2 for the FA and 45° for the angle between two contiguous eigenvectors. The three-dimensional fiber tracts were then superimposed on axial DWI.

### 2.4. Clinical Evaluation

During each visit, behavioral assessments were performed by clinicians. The Fugl-Meyer (FM) scale and Barthel index (BI) were measured to evaluate the patient's motor deficits. All three patients showed some motor function recovery at the time of the third DTI scan (1 month after onset).

## 3. Results

### 3.1. Clinical Data

The demographics and the evolution of motor function from baseline and throughout the 30 days of recovery in the three patients are summarized in Table 1. Patient 1 suffered from a left dorsolateral medullary infarction presenting with moderate right hemiparesis. Patient 2 had a smaller infarct in the right dorsolateral medullary with mild left hemiparesis. Both patients 1 and 2 had generally excellent motor recovery without disability (FM and BI score of 100) after 14 days. Patient 3 had an infarct of the left ventral medulla and deficits of the right upper extremity remaining after 30 days (FM score of 83.3 and BI score of 85).

### 3.2. Fractional Anisotropy

Table 2 shows the dynamic changes in the FA values of the infarct on the ipsilateral and contralateral sides of the CST from day 7 to day 30 in all three patients. Compared with the matched regions on the contralateral side, the FA values in the medulla, cerebral peduncle, and internal capsule on the infarct side all slightly decreased across the three time points. Of the three patients, patient 3 had the most obvious decrease in rFA values. The rFA values of the medulla decreased from 0.849 to 0.734 during the 30 days. In patients 1 and 2, the rFA values of the CST showed a slight decline but always stayed above 0.92.

### 3.3. Diffusion Tensor Tractography

**Figure 1:**
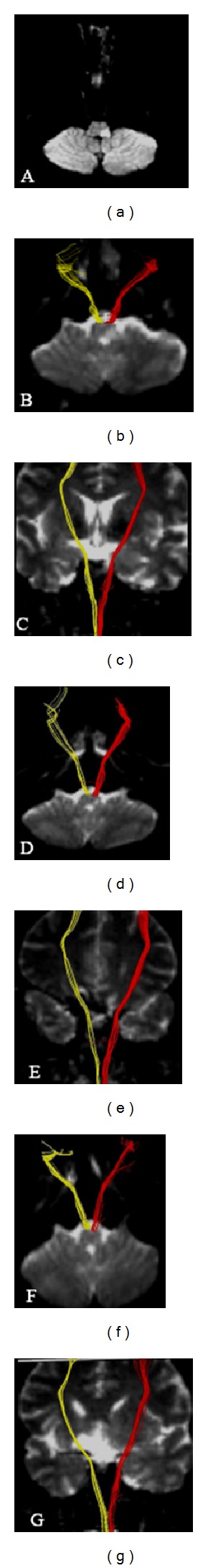
(a) Diffusion weighted imaging showed an infarct in the left dorsolateral medulla on day 3 after onset; (b)–(g) diffusion tensor tractography showed axial and coronary corticospinal tract ((b)–(c) on day 3; (d)-(e) on day 14; (f)-(g) on day 30). Red for the infarct side; yellow for the contralateral side. The integrity of the corticospinal tract was preserved around the infarct.

**Figure 2:**
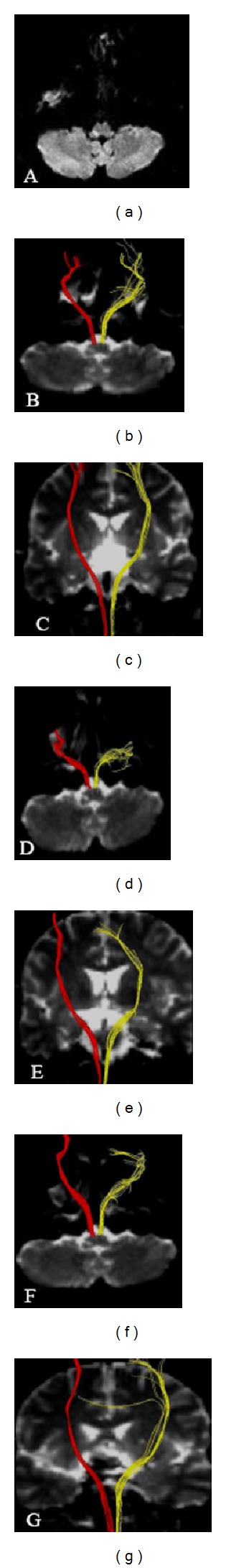
(a) Diffusion weighted imaging showed an infarct in the right dorsolateral medulla on day 5 after onset; (b)–(g) diffusion tensor tractography showed axial and coronary corticospinal tract ((b)-(c) on day 5; (d)-(e) on day 14; (f)-(g) on day 30). Red for the infarct side; yellow for the contralateral side. The integrity of the corticospinal tract was preserved around the infarct.

**Figure 3:**
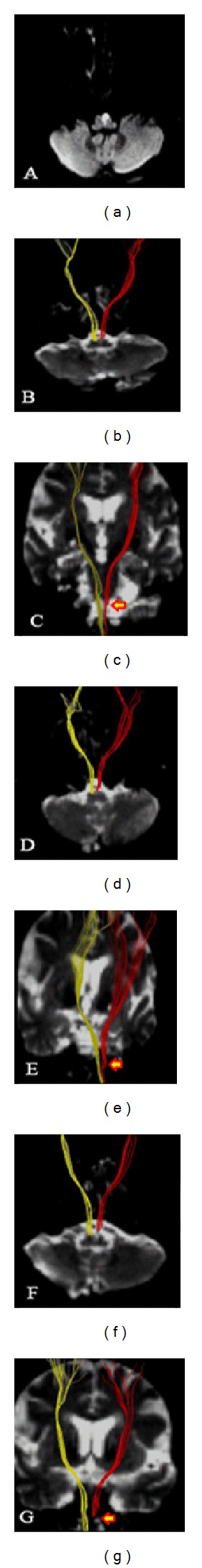
(a) Diffusion weighted imaging showed an infarct in the left ventral medulla on day 4 after onset; (b)–(g) diffusion tensor tractography showed axial and coronary corticospinal tract ((b)-(c) on day 4; (d)-(e) on day 14; (f)-(g) on day 30). Red for the infarct side; yellow for the contralateral side. The corticospinal tract was mostly interrupted by the infarct (arrow).

The DTTs for the CSTs of the unaffected hemispheres in the patients originated from the primary sensorimotor cortex and then descended along the known CST pathway. The CST descended from the affected hemisphere traveling via the anterior areas (patients 1 and 2) just around the infarct on the first DTT (Figures 1 and 2). The integrity of the CST was preserved around the infarct, just slightly compressed. On the followup DTTs, the CSTs of the affected hemisphere passed through the same respective spared periinfarct areas in these two patients. However, the left CST descended from the affected hemisphere traveling via the anterolateral around the infarct on the first DTT in patient 3 (Figure 3). The affected CST was partly disrupted below the medulla infarct. At day 14, the affected tracts were mostly interrupted by the infarct.

## 4. Discussion

In this study, we investigated the motor recovery mechanism in patients with unilateral medullary infarction who showed motor recovery after the onset of hemiparesis. The results revealed the deterioration of the CST associated with the motor outcome in patients. Differential patterns of recovery were found in the three patients. The main reason that the patients presented good motor recovery can be explained by the slight decline in the FA values and the maintenance of the integrity of the ipsilateral CST. The patients had shown severe paralysis of the right upper extremity at the onset of stroke; motor function recovered slowly over 1 month. The study suggests that the patients had poor motor outcomes in the affected extremities for the following reasons. First, low FA values were found in the affected CST compared with contralateral tracts. Second, DTTs demonstrated that a majority of the fiber tracts in the affected medulla were damaged.

We found that the FA values in the medulla, cerebral peduncle, and internal capsule on the infarct side all decreased across the three scan times. The size of medulla is small. Therefore the unilateral infarction and edema of medulla may affect bilateral corticospinal tract. The FA values in the medulla, cerebral peduncle, and internal capsule were found decreased on both sides. In patient 3, the ventral medulla infarction was relatively large, so the FA values decreased more obvious. In patients 1 and 2, the FA values of the affected CST showed a slight decline, although the fiber was spared. Patient 3 had the most obvious decrease in FA values along the affected CST and the rFA values of the medulla decreased from 0.849 to 0.734 during the 30 days. Because the blood supply to the internal capsule and cerebral peduncle differs from those in the medulla, our findings cannot be explained by ischemia. Previous studies reported that the FA reduction reflects deterioration of axonal integrity leading to axonal loss and Wallerian degeneration (WD) [6, 7]. Compared with conventional MRI imaging, such as that with T2 and FLAIR sequences, FA values are an early marker of axonal degeneration. In addition, several longitudinal studies of DTI in patients with cerebral infarction demonstrated WD of the pyramidal tract. Therefore, the changes in the FA values in the medulla and along the CST in our study were due to axonal injury and WD. The mechanism of retrograde secondary damage in the cerebral peduncle and internal capsule is consistent with that of anterograde WD.

Our study confirms that DTT can provide important clues to the spatial relationship between infarcts and the CST in stroke patients. Therefore, it can be used to predict motor outcomes. The affected CST descended running via the anterior areas just around the infarct on DTTs in patients 1 and 2. The integrity of the CST was preserved. The motor recovery could be attributed to periinfarct spared CST or the resolution of the transient edema around the lesion. In patient 3, the affected CST was partly disrupted and the motor function slowly recovered. Thus, CST integrity plays an important role during the recovery of motor function. Previous studies have suggested that motor function can be controlled well only by a part of the CST [8]. In this study, it seems that patients' motor function was controlled via the spared periinfarct CST, also in the medulla. However, patients with more CST involvement tend to have poorer prognosis. So far, many studies on the state of the CST or motor recovery mechanisms at the subcortical level have been reported [9–12]. However, to our knowledge, only one study has used DTI to investigate patients with medulla infarct [13]. Jang et al. reported one patient whose motor functions of the affected extremities rapidly recovered to a normal state over the 4 months following stroke onset. The integrity of the CST was shown to be spared in the anterior portion of the medulla infarct using DTT.

In conclusion, we demonstrated that DTI could detect the involvement of and changes in the CST in patients with medulla infarct during motor recovery. The degree of degeneration and the spared periinfarct CST compensation may be an important mechanism of motor recovery. However, this present study is admittedly limited because only three patients were involved. Therefore, our findings need to be further verified in studies with larger case numbers and combined DTI and functional MRI techniques.

## Figures and Tables

**Table 1:** Patient demographic and clinical data.

Patient	Sex/age	Symptoms	FM			BI		
			<7 d	14 d	30 d	<7 d	14 d	30 d
1	M/55	R hemiparesis	98.5	100	100	95	100	100
2	M/54	L hemiparesis	97	100	100	98	100	100
3	F/74	R hemiplegia	36.4	70.5	83.3	50	80	85

M = male; F = female; L = left; R = right; FM, Fugl-Meyer; BI, Barthel index.

**Table 2:** FA Values of corticospinal tract in the patients with medullary infarction.

Patient		Medulla			Cerebral peduncle			Internal capsule		
		<7 d	14 d	30 d	<7 d	14 d	30 d	<7 d	14 d	30 d
1	Ipsi	0.528 ± 0.217	0.546 ± 0.042	0.557 ± 0.163	0.744 ± 0.107	0.688 ± 0.118	0.799 ± 0.119	0.729 ± 0.080	0.701 ± 0.043	0.700 ± 0.024
	Contra	0.544 ± 0.152	0.554 ± 0.158	0.577 ± 0.088	0.758 ± 0.124	0.695 ± 0.053	0.811 ± 0.012	0.740 ± 0.046	0.736 ± 0.022	0.721 ± 0.035
	rFA	0.971	0.986	0.965	0.982	0.990	0.985	0.985	0.952	0.971

2	Ipsi	0.646 ± 0.131	0.622 ± 0.208	0.655 ± 0.221	0.784 ± 0.011	0.827 ± 0.066	0.775 ± 130.1	0.793 ± 0.027	0.798 ± 0.022	0.768 ± 0.076
	Contra	0.695 ± 0.169	0.651 ± 0.167	0.684 ± 0.013	0.801 ± 0.042	0.857 ± 0.051	0.799 ± 0.056	0.806 ± 0.114	0.863 ± 0.054	0.807 ± 0.101
	rFA	0.9300	0.955	0.958	0.979	0.965	0.970	0.984	0.925	0.951

3	Ipsi	0.331 ± 0.093	0.404 ± 0.061	0.441 ± 0.082	0.767 ± 0.050	0.736 ± 0.032	0.773 ± 0.060	0.774 ± 0.069	0.769 ± 0.107	0.781 ± 0.086
	Contra	0.390 ± 0.110	0.536 ± 0.106	0.601 ± 0.299	0.832 ± 0.043	0.852 ± 0.083	0.868 ± 0.071	0.780 ± 0.080	0.778 ± 0.092	0.786 ± 0.120
	rFA	0.849	0.754	0.734	0.922	0.864	0.891	0.992	0.988	0.994
